# A 2D Markerless Gait Analysis Methodology: Validation on Healthy Subjects

**DOI:** 10.1155/2015/186780

**Published:** 2015-04-30

**Authors:** Andrea Castelli, Gabriele Paolini, Andrea Cereatti, Ugo Della Croce

**Affiliations:** ^1^Department of Information Engineering, Political Sciences and Communication Sciences, University of Sassari, 07100 Sassari, Italy; ^2^Interuniversity Centre of Bioengineering of the Human Neuromusculoskeletal System, Sassari, Italy

## Abstract

A 2D markerless technique is proposed to perform lower limb sagittal plane kinematic analysis using a single video camera. A subject-specific, multisegmental model of the lower limb was calibrated with the subject in an upright standing position. Ankle socks and underwear garments were used to track the feet and pelvis segments, whereas shank and thigh segments were tracked by means of reference points identified on the model. The method was validated against a marker based clinical gait model. The accuracy of the spatiotemporal parameters estimation was found suitable for clinical use (errors between 1% and 3% of the corresponding true values). Comparison analysis of the kinematics patterns obtained with the two systems revealed high correlation for all the joints (0.82 < *R* ^2^ < 0.99). Differences between the joint kinematics estimates ranged from 3.9 deg to 6.1 deg for the hip, from 2.7 deg to 4.4 deg for the knee, and from 3.0 deg to 4.7 deg for the ankle. The proposed technique allows a quantitative assessment of the lower limb motion in the sagittal plane, simplifying the experimental setup and reducing the cost with respect to traditional marker based gait analysis protocols.

## 1. Introduction

Three-dimensional (3D) marker-based clinical gait analysis is generally recognized to play an important role in the assessment, therapy planning, and evaluation of gait related disorders [1]. It is performed by attaching physical markers on the skin of the subject and recording their position via multiple cameras. To date, optoelectronic stereophotogrammetric systems represent the most accurate technology for the assessment of joint kinematics [2]. While the 3D characterization of motion represents the standard for clinical gait analysis laboratories and research environments, 3D gait analysis remains underused in ambulatory environments due to the costs, time, and technical requirements of this technology.

Multicamera, video-based markerless (ML) systems can represent a promising alternative to 3D marker-based systems [3–5]. In fact, the use of ML techniques does not require the application of fixtures on the skin of the patients [1], making the experimental sessions faster and simpler (e.g., do not have to worry about markers falling off during the sessions) [1]. 3D ML motion capture techniques have been extensively presented in [5–9] for different types of applications, including clinical gait analysis and biomechanics. However, 3D ML approaches, similarly to 3D marker-based techniques, require the use of multiple cameras [5], specific calibration procedures, time synchronization between cameras, and a considerable dedicated space. Furthermore, the number of cameras and their high image resolution dramatically increase the computing time and resources required.

When a 3D analysis is not strictly required, a simpler 2D analysis on the sagittal plane could be successfully used to quantify gait and to address specific clinical questions [10, 11]. A single-camera approach is sufficient for the description of gait in 2D and allows for a simplified experimental setup reducing the space needed for the equipment, the number of cameras, and the costs associated. Video recording in combination with observational gait evaluation scales [11] is commonly employed to perform visual and qualitative gait analysis. In this regard, a methodology to quantitatively assess joint kinematics from the video footage can provide added value to the patient care with no extra resources involved.

Single-camera ML techniques have been mainly developed for motion recognition and classification [12–19]. To the best of our knowledge, only a paucity of studies have proposed ML methods for the estimation of the lower limb joint kinematics for clinical evaluations [20–24]. However, in the abovementioned studies, the analyses were either limited to a single joint [20, 21], or they lacked a validation against a clinically accepted gold standard [22, 23]. These limitations hampered the wide spread use of such techniques in clinical settings [4, 7, 9].

In this study, we present a novel 2D, model-based, ML method for clinical gait analysis using a single camera: the SEGMARK method. It provides unilateral joint kinematics of hip, knee, and ankle in the sagittal plane, along with the estimation of gait events and spatiotemporal parameters. The method uses garments (i.e., socks and underwear) worn by the subjects as segmental markers to track the pelvis and feet segments. The segments' model templates and the relevant anatomical coordinate systems are calibrated in a static reference image from anatomical landmarks manually identified by an operator. The method applicability was tested and the performance evaluation was carried out on ten healthy subjects walking at three different speeds using an optoelectronic marker-based system as gold standard.

## 2. Materials and Methods

### 2.1. Experimental Protocol and Setup

Ten healthy subjects (males, age 33 ± 3 y.o.), wearing only homogeneously coloured (white) and adherent ankle socks and underwear, were asked to walk at comfortable, slow, and fast speed along a straight 8-meter walkway. An RGB video camera (Vicon Bonita Video 720c, 1280 × 720 p, 50 fps) was positioned laterally to the walkway. A homogenous blue background was placed opposite to the camera. To prevent blurred images, the exposure time was set to a low value (5 ms), and the illumination level was set accordingly. The image coordinate system (CS_I_) of the video camera was aligned to the sagittal plane, identified by the direction of progression and the vertical direction.

Three trials per subject were captured for each gait speed. The starting line was set so that the foot in the foreground could enter the field of view first and hit the ground when fully visible. A static reference image, with the subject in an upright standing position, centered in the field of view of the camera, was captured prior to each experimental session. The subjects were then asked to walk along a line drawn on the floor, placed at a known distance from the image plane, identical to the distance between the camera and the subject during the static reference image acquisition. For validation purposes, 3D marker-based data, synchronous with the video data, was captured at 50 fps using a 6-camera stereophotogrammetric system (Vicon T20). Retroreflective spherical markers (14 mm diameter) were attached to the subjects according to the Davis model [26] provided by the Vicon Nexus software (*Plug in Gait*).

### 2.2. Image Preprocessing

Camera lens distortion was corrected using the Heikkilä undistortion algorithm [27]. The spatial mapping of the camera image was determined by associating the measured foot length to the foot segmental marker length expressed in pixels, from the static reference image (1 pixel ≈ 1 mm).

**Figure 1:**
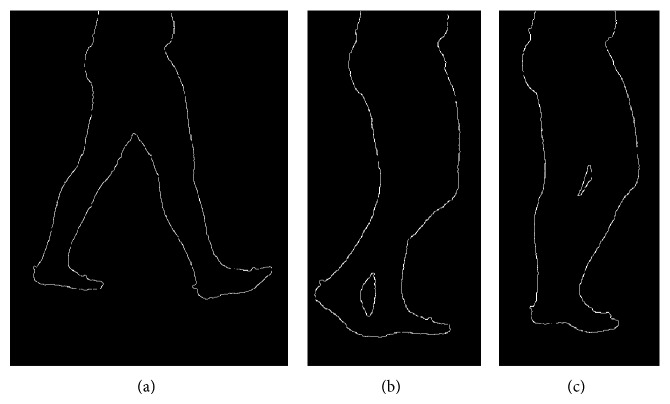
Contour extraction and silhouette contour deformation for three different gait cycle percentages. It can be noticed that in cases (b) and (c) there is an overlap between the foreground and background legs that prevents the identification of the correspondent boundaries of the segments.

To separate the moving subject from the background, a segmentation procedure based on background subtraction in the HSV color space was applied [6]. The underwear and ankle socks were extracted using a white color filter and used as segmental markers. An automatic labelling process to identify the segmental markers was performed. The pelvis segmental marker was identified as the group of white pixels with higher vertical coordinates in the CS_I_. The feet segmental markers were identified and tracked using the predicted positions of their centroids, based on their velocity at the previous two frames. Canny's edge operator [28] was used to obtain the silhouette (Figure 1) and the segmental markers contours.

### 2.3. Cycle Segmentation and Gait Parameters Determination

Heel strike and toe off events were automatically estimated using a method originally developed for marker-based systems [29] and adapted to our ML method. Per each time frame, the centroids of the pelvis and both feet were determined. The gait events (heel strike and toe off) instants were determined when the maximum horizontal relative distance between the pelvis and the foot centroids is achieved.

An expert operator manually identified the same gait events, using the video footage and the heel and toe 3D marker trajectories as reference. The following spatial and temporal parameters were then calculated for both ML and marker-based data: cadence, walking speed, stride time, and stride length. The estimated parameters were compared for validation purposes.

### 2.4. Model Calibration

**Figure 2:**
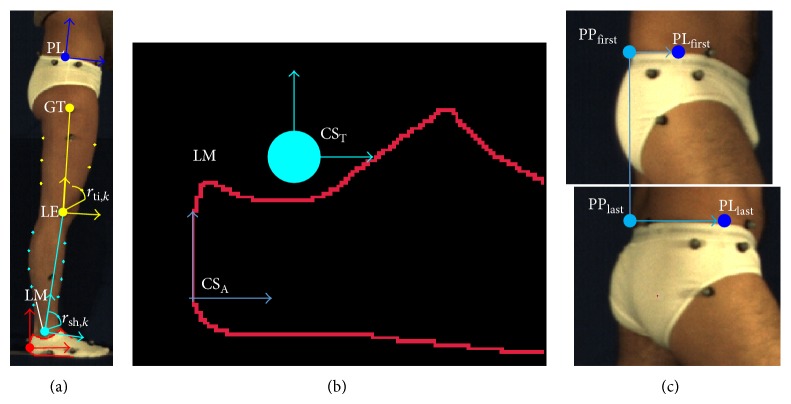
(a) Anatomical landmarks and anatomical coordinate systems (CS_A_) for the segments analyzed; femur CS_A_ (yellow axes) and femur reference points (yellow points) identified by the yellow arcs; tibia CS_A_ (cyan axes) and tibia reference points (cyan points) identified by the cyan arcs. (b) Foot CS_A_ and model template. (c) Double anatomical calibration of the most lateral point (PL) in the first and last frame of the gait cycle.

A subject-specific, multisegmental model of the lower limb, was used to track the segments and to compute the relevant joint kinematics. The model is made of four segments (foot, tibia, femur, and pelvis) connected by hinges. The position of the following anatomical landmarks was manually identified by the operator in the static reference image: lateral malleolus (LM), lateral femoral epicondyle (LE), and greater trochanter (GT) (Figure 2(a)).

The foot model template was defined as the posterior half of the foot segmental marker contour. The anatomical coordinate system (CS_A_) of the foot was defined on the foot model template, with the positive *x*-axis coincident with the line fitting the lower-posterior contour and oriented towards the toes. The origin was made to coincide with the most posterior point of the foot segmental marker contour. A technical coordinate system (CS_T_) was defined with the *x*-axis coinciding with the corresponding axis of the CS_I_ and centred in LM (Figure 2(b)). The transformation matrix between the CS_T_ and CS_A_ of the foot was computed ^fCS_A_^ **T**_fCS_T__.

The CS_A_ of the tibia was defined with the *y*-axis joining LM with LE (origin on LM). The tibia model template was defined based on ten reference points identified on the silhouette as the intersections between the shank contour and the circles of radius *r*_sh,*k*_, centered in LM (Figure 2(a)). The length of the imposed radii was chosen so that the reference points would fall within the middle portion of the shank segment (between the 25% and 75% of the segment length). This avoided the reference points to fall on the portions of the segment adjacent to the joints. These areas are, in fact, subject to a larger soft tissue deformation during gait [2]. The tibia CS_T_ was defined with the *x*-axis parallel to the *x*-axis of the CS_I_ and centred in the centroid of the tibia reference points. The transformation matrix between CS_T_ and CS_A_ of the tibia was computed ^tibCS_A_^ **T**_tibCS_T__ (Figure 2(a)). LM and LE positions and the tibia reference points were then expressed in the tibia CS_T_ (^tibCS_T_^ **p**_*k*_^0^, *k* = 1,…, 10).

The CS_A_ of the femur was defined with the *y*-axis joining LE with GT (origin on LE). The femur model template was defined based on six reference points identified on the silhouette as the intersections between the thigh contour and the circles of radius *r*_th,*k*_, centered in LE (Figure 2(a)). The femur CS_T_ was defined with the *x*-axis parallel to the *x*-axis of the CS_I_ and centred in the centroid of the thigh reference points. The transformation matrix between the CS_T_ and CS_A_ of the femur was computed ^femCS_A_^ **T**_femCS_T__ (Figure 2(a)). LE and GT positions and the femur reference points were then expressed in the femur CS_T_ (^femCS_T_^ **p**_*k*_^0^, *k* = 1,…, 6).

The pelvis CS_A_ origin was set in the most lateral point (PL) of the pelvis segmental marker upper contour (Figure 2(a)). The *x*-axis was oriented as the line fitting the portion of the pelvis segmental marker upper contour defined from PL ± 20 pixels and pointing towards the direction of progression. Due to both parallax effect and pelvis axial rotation during gait, the shape of the pelvis segmental marker changes remarkably throughout the gait cycle (Figure 2(c)). To improve the quality of the PL tracking process, a double calibration approach was implemented. At the first and last frames of the gait cycle, the PL positions were manually identified by the operator (PL_first_, PL_last_) whereas the positions of the most posterior point (PP_first_ and PP_last_) of the pelvis upper contour were automatically identified. The horizontal distances between PL_first_ and PP_first_ (*d*_*x*,first_) and between PL_last_ and PP_last_ (*d*_*x*,last_) were calculated and the incremental frame by frame variation Δ was computed according to

(1) $$\begin{array}{c} \Delta =\frac{\left|d_{x,\text{last}}-d_{x,\text{first}}\right|}{M}, \end{array}$$

with *M* representing the number of frames.

The incremental frame variation was then used to compute the distance *d*_*x*,*i*_ between the points PP and PL in correspondence of the *i*th frame:

(2) $$\begin{array}{c} d_{x,i}=d_{x,\text{first}}+\left(\;\Delta \cdot i\;\right). \end{array}$$

The position of PL at the *i*th instant (PL_*i*_) was determined from the automatically detected position of PP at the same time instant (PP_*i*_):

(3) $$\begin{array}{c} {\text{PL}}_{i}={\text{PP}}_{i}+d_{x,i}. \end{array}$$

### 2.5. Dynamic Processing

The dynamic trials were processed using a bottom-up tracking approach, starting from the foot and moving up the chain. The foot was tracked using an iterative contour subtraction matching technique between the contour at the *i*th frame and the template foot contour. At the *i*th frame, the foot CS_T_ was rotated and translated around the prediction of the LM position based on its velocity estimated in the previous two frames. To make the process more efficient, the rotational span limits were defined based on the gait cycle phase: ±10 degrees during the stance phase and ±30 degrees during the swing phase. The transformation matrix ^fCS_T_^ **T**(*i*)_fCS_I__ between the foot CS_T_ and the CS_I_ was determined by maximizing the superimposition between the foot template and the foot contour at the *i*th frame (i.e., the minimum number of active pixels resulting from the image subtraction). The transformation matrix between the foot CS_A_ and the CS_I_ was computed as

(4) TifCSIfCSA=TifCSTfCSA·TifCSIfCST.

**Figure 3:**
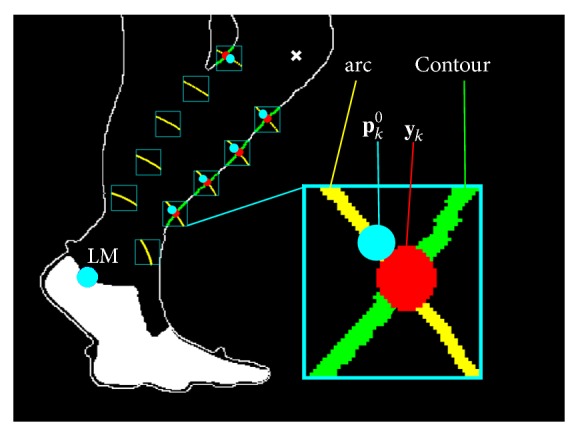
Tibia reference points detection. These points are identified on the silhouette as the intersections between the shank contour and the circles of radius *r*_sh,*k*_, centered in LM. LM (cyan circle); predicted LE (white cross); magnified: arc of circumference of radius *r*_sh,*k*_ (yellow curve); **y**_*k*_: reference point detected in the current frame (red circle); **p**_*k*_^0^: template reference point after fitting (cyan circle); silhouette contour line (contour).

Due to leg superimposition and soft tissues deformation, the silhouette contours of the shank and thigh change during the gait cycle, making the tracking difficult (Figure 1). To overcome this issue, the following procedure was adopted for the tibia. At the *i*th frame, a registration of first approximation between the tibia CS_T_ and the CS_I_ was carried out using the position of LM and the prediction of LE. An approximated estimate of the tibia reference points positions with respect to the CS_I_ was then obtained and a 10 × 10 pixels region of interest was created around each point. The final reference position vectors ^CS_I_^ **y**_*k*_ of the tibia were detected as the intersection, where available, between the portion of the shank contour included in the region of interest, and the circle of radius *r*_tib,*k*_. The transformation matrix ^tibCS_T_^ **T**(*i*)_tibCS_I__ between the CS_I_ and CS_T_ was determined using a Singular Value Decomposition procedure [25] between the position vectors ^tibCS_T_^ **p**_*k*_^0^ and the corresponding points ^tibCS_I_^ **y**_0_. Due to leg superimposition, the number of points ^tibCS_T_^ **p**_*k*_^0^ involved in the fitting procedure varied according to the number of intersections ^tibCS_I_^ **y**_0_ available (Figure 3). The transformation matrix between the tibia CS_A_ and the CS_I_ was computed as

(5) TitibCSItibCSA=TitibCSTtibCSA·TitibCSItibCST.

An identical procedure was employed to determine the transformation between the CS_I_ and CS_A_ of the femur ^femCS_A_^ **T**(*i*)_femCS_I__ at the *i*th frame.

From the relevant transformation matrices, the joint angles between the pelvis and the femur (hip flex/ext.), between the femur and the tibia (knee joint flex/ext.), and between the tibia and the foot (ankle joint flex/ext.) were computed.

### 2.6. Data Analysis

For each gait speed, the accuracy of the spatiotemporal gait parameters estimated by the ML approach was assessed in terms of the mean absolute error (MAE) and MAE% over trials and subjects (3 × 10 trials). Both the ML and marker-based angular kinematic curves were filtered using a fourth-order Butterworth filter (cut-off frequency at 10 Hz). The sagittal angular kinematics produced by the* Plug in Gait* protocol was used as gold standard [26].

The kinematic variables were time-normalized to the gait cycle. Furthermore, for each gait trial and each joint, the average root-mean-square deviation (RMSD) value between the joint kinematic curves estimated by the ML method and the gold standard were computed over the gait cycle and averaged across trials and subjects. The similarity between the curves provided by the ML method and the gold standard was assessed using the linear-fit method proposed by Iosa and colleagues [30]. The kinematic curves produced by the ML system were plotted versus those produced by the gold standard, and the coefficients of the linear interpolation were used to compare the curves in terms of shape similarity (*R* ^2^), amplitude (angular coefficient, *A*0), and offset (constant term, *A*1). The average of *A*0 and *A*1 across trials and subjects was calculated for each gait speed.

## 3. Results

The results relative to the spatiotemporal gait parameters are shown in Table 1. For all parameters and gait speeds, the errors were between 1% and 3% of the corresponding true values.

Results relative to the joint kinematics are shown in Table 2. Average RMSD values, over trials and subjects, ranged from 3.9 deg to 6.1 deg for the hip, from 2.7 deg to 4.4 deg for the knee, from 3.0 deg to 4.7 deg for the ankle, and from 2.8 deg to 3.8 deg for the pelvic tilt.

**Figure 4:**
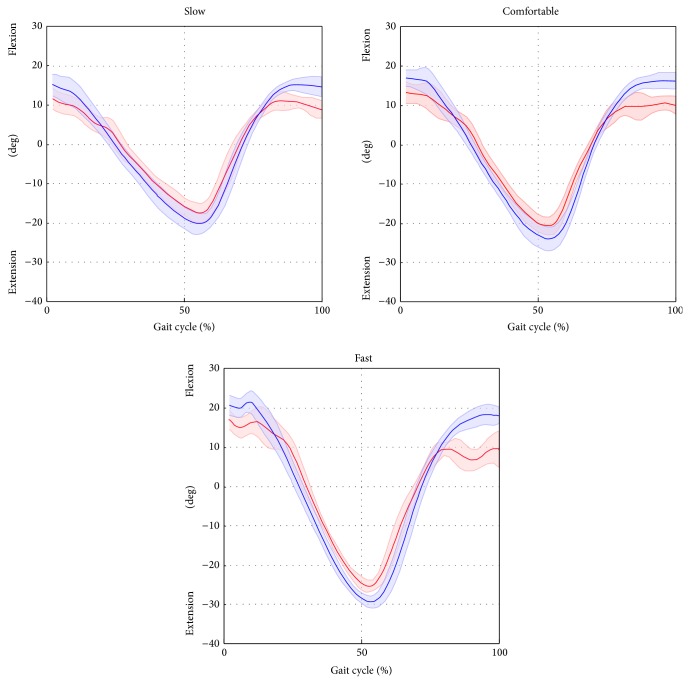
Hip flexion/extension averaged over subjects and trials for the selected gait speed (average: solid lines; SD: shaded area; red = ML; blue =* Plug in Gait* protocol).

**Figure 5:**
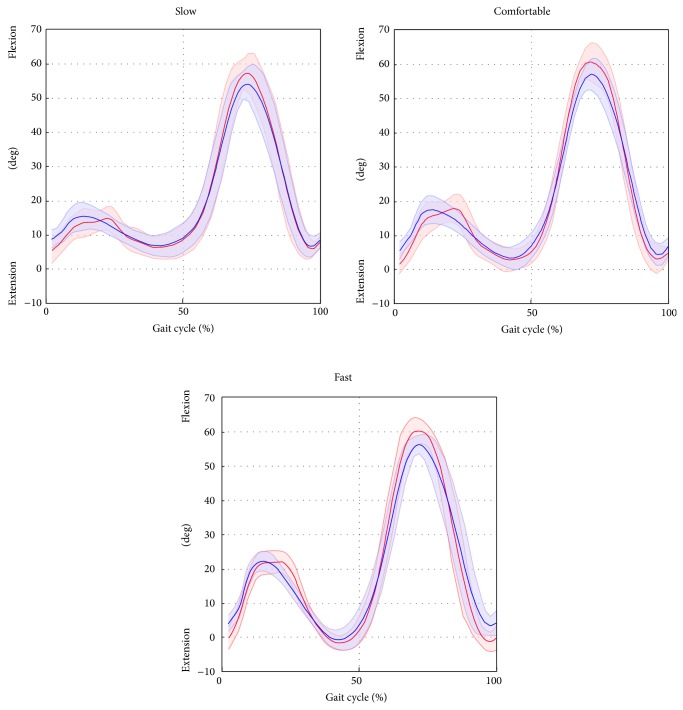
Knee flexion/extension averaged over subjects and trials for the selected gait speed (average: solid lines; SD: shaded area; red = ML; blue =* Plug in Gait* protocol).

**Figure 6:**
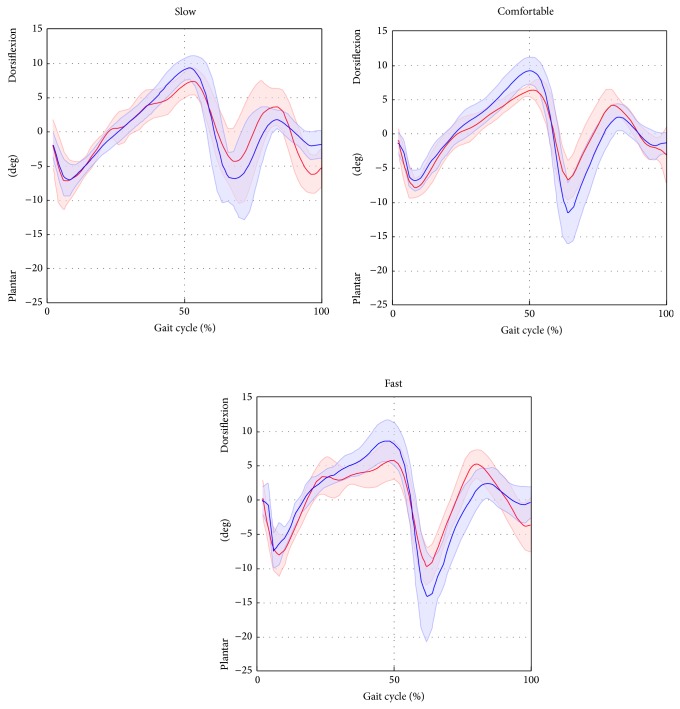
Ankle plantar/dorsiflexion averaged over subjects and trials for the selected gait speed (average: solid lines; SD: shaded area; red = ML; blue =* Plug in Gait* protocol).

**Figure 7:**
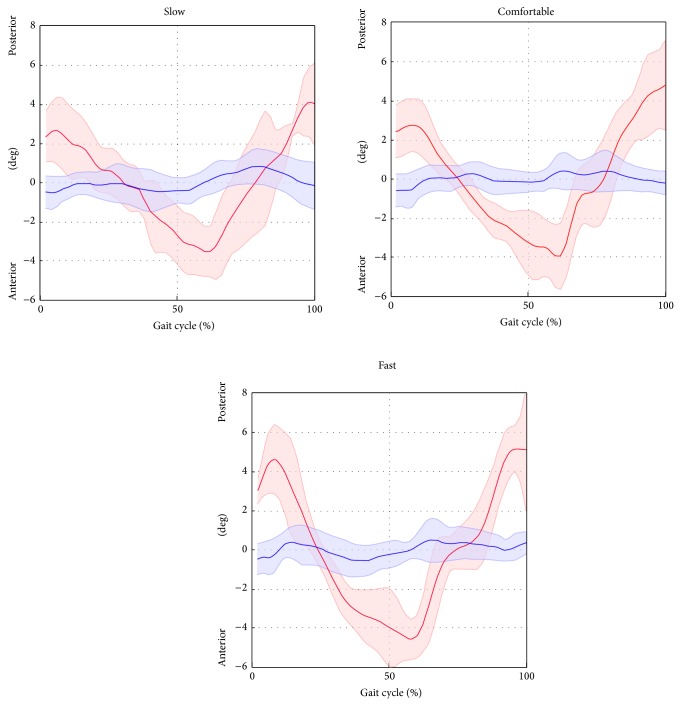
Pelvic tilt averaged over subjects and trials for the selected gait speed (average: solid lines; SD: shaded area; red = ML; blue =* Plug in Gait* protocol).

The results of the linear-fit method (*R* ^2^) highlighted excellent correlation (from 0.96 to 0.99 for all gait speeds) for hip and knee joint kinematics. The ankle kinematics showed strong correlation (from 0.82 to 0.87 for all gait speed). Conversely, the pelvic tilt showed no correlation. The hip and ankle joint kinematics were underestimated in terms of amplitude (*A*0 ranged from 0.73 to 0.76 and from 0.82 to 0.87 for the hip and ankle, resp.), whereas the knee kinematics were overestimated (*A*0 from 1.08 to 1.11). The values of the offset *A*1 were consistent amongst the different gait speeds (maximum difference of 2.4 deg for the hip joint, across all gait velocities) and ranged from −0.1 deg to 2.3 deg, from −7.7 deg to −6.4 deg, and from −4.7 deg to −3.3 deg for the hip, knee, and ankle, respectively. The joint kinematics and pelvic tilt curves, averaged over trials and subjects and normalised to the gait cycle, are reported in Figures 4, 5, 6, and 7.

## 4. Discussion and Conclusion

The aim of this study is to implement and validate a ML method based on the use of a single RGB camera for lower limb clinical gait analysis (SEGMARK method). The estimated quantities consist of hip, knee, and ankle joint kinematics in the sagittal plane, pelvic tilt, and spatiotemporal parameters of the foreground limb. The SEGMARK method is based on the extraction of the lower limbs silhouette and the use of garment segmental markers for the tracking of the foot and pelvis.

Key factors of the proposed method are the use of anatomically based coordinate systems for the joint kinematics description, the automatic management of the superimposition between the foreground and background legs, and a double calibration procedure for the pelvis tracking.

For a clinically meaningful description of the joint kinematics, it is required, for each bone segment, to define a coordinate system based on the repeatable identification of anatomical landmarks [31]. In our method, the anatomical calibration is performed by an operator on the static reference image, by clicking on the relevant image pixels. Although it might sound as a limitation, the manual anatomical landmarks calibration is a desired feature because it allows the operator to have full control on the model definition.

When using a single RGB camera for recording gait, the contours of the foreground and background legs are projected onto the image plane, and their superimposition makes difficult the tracking of the femur and tibia segments during specific gait cycle phases (Figure 1). To overcome this problem, the model templates of the femur and tibia segments were matched, for each frame, to an adaptable set of target points automatically selected from the thigh and shank contours.

The use of a silhouette-based approach implies that no information related to the pixel greyscale intensity or color values are exploited, except for the background subtraction procedure [7, 8]. This should make the method less sensitive to change in light conditions or cameras specifications [7, 8].

The accuracy with which the spatiotemporal parameters are estimated (Table 1) is slightly better than other ML methods [14, 15]. Furthermore, the percentage error was always lower than 3%. The errors associated to the gait temporal parameter (stride time) estimation were less than 0.02 s, for all gait speeds.

The kinematic quantities estimated by the SEGMARK method and those obtained using a clinically validated gait analysis protocol were compared in terms of root-mean-square deviation (RMSD), shape (*R* ^2^), amplitude (*A*0), and offset (*A*1) (Table 2). In this respect, it is worth noting that the differences found are not entirely due to errors in the joint kinematics estimates, but also to the different definitions used for the CS_A_ and the different angular conventions adopted (2D angles versus 3D Euler angles).

Overall, the RMSD values ranged between 2.7 and 6.1 deg across joints and gait speeds. The smallest RMSD values were found for the knee joint kinematics, followed by the hip and ankle joints kinematics. We reckon that the differences at the hip angle between the SEGMARK method and the Plug in Gait model were mainly due to inaccuracies in the pelvis tracking, which proved to be a critical aspect of the single-camera technique proposed. This is mainly due to intrinsic difficulties associated to the pelvis tracking, such as the small range of motion in the sagittal plane during gait (<5 deg), the wider range of motion in the frontal and horizontal planes, and the difficulty to isolate the pelvis from the lower trunk. The differences in the ankle joint kinematics observed between the SEGMARK method and the* Plug in Gait* model (Table 2) can be probably ascribed to the 2D tracking of the foot segment. In fact, the SEGMARK method cannot account for the foot motion in the transverse plane, leading to an underestimation of the ankle joint motion amplitude (*A*0 varied from 0.82 to 0.68 for increasing gait speed). The waveform similarity analysis highlighted that the amplitude of the knee joint angle was consistently overestimated (*A*0 equals to 1.11 for comfortable speed) whereas the hip and ankle joint kinematic curves were consistently underestimated (*A*0 equals to 0.76 and 0.74 for hip and ankle, resp., for comfortable speed). This can be explained by the consistent overestimation of the pelvis motion, which was in phase with the femur angular displacement, combined with the underestimation of the foot motion in the sagittal plane. An increase of the gait speed showed a negative effect on the accuracy of the kinematics estimation for all the joints due to the significantly smaller number of frames recorded at the fast gait (≈40 frames) compared to the slow gait (≈80 frames). This result underlines the importance of a sufficiently high frame rate when recording the video footage.

To our knowledge, amongst the 2D ML methods for clinical gait analysis proposed in the literature, just a few use anatomically relevant points to define the CS_A_ [22, 32, 33]. Goffredo and colleagues [22] used an approach based on skeletonisation and obtained the proportions of the human body segments from anatomical studies [34]. This approach, although completely automatic, neglects the subject-specific characteristics, possibly leading to joint angles estimation inaccuracies. Other recent ML validation studies rely upon the built-in algorithm of the Microsoft Kinect to identify joint centres [33, 35], which are therefore not based on anatomical information, making the use of such techniques questionable for clinical applications. Another important limitation of the 2D ML methods previously proposed is the lack of a systematic validation via a well-established gold standard for clinical gait analysis applications [13–19]. To our knowledge, only the work presented by Majernik [32] compared the joint kinematics with the sagittal plane joint angles produced by a simultaneously captured 3D marker-based protocol. However, neither reference to the specific clinical gait analysis protocol used nor a quantitative assessment of the method performance is reported. Similarly, Goffredo and colleagues [22] only performed a qualitative validation of their method by comparing the estimated joint kinematics to standard patterns taken from the literature. Moreover, none of the abovementioned works described the procedure used to track the pelvis, which is critical for the correct estimation of the hip joint angle.

Interestingly, when comparing our results with the joint sagittal kinematics obtained from more complex 3D ML techniques, we found errors either comparable or smaller. Specifically, Sandau et al. [3] reported a RMSD of 2.6 deg for the hip, 3.5 deg for the knee, and 2.5 deg for the ankle, which are comparable with the RMSD values reported in Table 2 for the comfortable gait speed. Ceseracciu et al. [4] reported a RMSD of 17.6 deg for the hip, 11.8 deg for the knee, and 7.2 deg for the ankle, sensibly higher than the values reported in this work. This further confirms the potential of the SEGMARK approach for the study of the lower limb motion in the sagittal plane for clinical applications.

This study has limitations, some of them inherent to the proposed ML technique, whereas others related to the experimental design of the study. First, the joint kinematics is only available for the limb in the foreground, while other authors managed to obtain a bilateral joint kinematics description using a single camera [22]. Therefore, to obtain kinematics from both sides, the subject has to walk in both directions of progression. Second, the segmentation procedure for the subject silhouette extraction takes advantage of a homogeneous blue background. This allows for optimal segmentation results, but it adds a constraint in the experimental setup. For those applications where the use of a uniform background is not acceptable, more advanced segmentation techniques can be employed [7–9]. Finally, the anatomical calibration procedure requires the operator to visually identify the anatomical landmarks in the static image, and this operation necessarily implies some repeatability errors which would need to be assessed in terms of inter and intra observer and inter and intrasubject variability [35]. As a future step, the present methodology will be applied and validated on pathological populations such as cerebral palsy children.

## Figures and Tables

**Table 1:** Gait spatiotemporal parameters. Mean absolute (MAE) and percentage errors of the cadence, walking speed, stride time, and stride length for different gait speeds (slow, comfortable, and fast).

	Slow speed		Comfortable speed		Fast speed	
	MAE	MAE%	MAE	MAE%	MAE	MAE%
Cadence (steps/min)	0.93	1%	1.34	1%	2.23	2%

Walking speed (m/s)	0.02	2%	0.03	3%	0.05	3%

Stride time (s)	0.01	1%	0.01	1%	0.02	2%

Stride length (m)	0.03	3%	0.04	3%	0.05	3%

**Table 2:** Lower limb joint and pelvis kinematics. The average root-mean-square deviation (RMSD) value between the joint kinematics curves estimated by the ML method and the gold standard are computed over the gait cycle and averaged across trials and subjects. The similarity between the curves obtained with the proposed ML method and the gold standard is assessed using the linear-fit method [25]. The coefficients of the linear interpolation were used to compare the curves in terms of shape similarity (*R* ^2^), amplitude (angular coefficient, *A*0), and offset (constant term, *A*1). The average of *A*0 and *A*1 across trials and subjects is calculated for each gait speed.

Speed	Hip				Knee				Ankle				Pelvis			
	RMSD (deg)	*R* ^2^	*A*0	*A*1 (deg)	RMSD (deg)	*R* ^2^	*A*0	*A*1 (deg)	RMSD (deg)	*R* ^2^	*A*0	*A*1 (deg)	RMSD (deg)	*R* ^2^	*A*0	*A*1 (deg)
Slow	3.9	.97	.76	1.02	2.7	.99	1.08	−6.36	3.2	.84	.82	−4.67	2.8	.06	.05	−3.04
Comfortable	4.8	.97	.76	−.09	3.6	.99	1.11	−7.71	3.0	.87	.74	−3.64	3.0	.01	.38	−6.71
Fast	6.1	.96	.73	2.31	4.4	.98	1.10	−6.47	4.7	.82	.68	−3.35	3.8	.18	−.72	−.19
